# Development and validation of a new patient-reported outcome measure for patients with pressure ulcers: the PU-QOL instrument

**DOI:** 10.1186/1477-7525-11-95

**Published:** 2013-06-13

**Authors:** Claudia Gorecki, Julia M Brown, Stefan Cano, Donna L Lamping, Michelle Briggs, Susanne Coleman, Carol Dealey, Elizabeth McGinnis, Andrea E Nelson, Nikki Stubbs, Lyn Wilson, Jane Nixon

**Affiliations:** 1 Clinical Trials Research Unit (CTRU), University of Leeds, Leeds LS2 9JT, UK; 2 Clinical Neurology Research Group, Peninsula College of Medicine and Dentistry, Plymouth, UK; 3 Department of Health Services Research and Teaching, London School of Hygiene & Tropical Medicine, London, UK; 4 School of Healthcare, University of Leeds, Leeds, UK; 5 University Hospital Birmingham NHS Foundation Trust, Birmingham, UK; 6 Tissue Viability Service, Leeds Teaching Hospitals NHS Trust, Leeds, UK; 7 Leeds Community Healthcare NHS Trust, Leeds, UK

**Keywords:** Pressure ulcer, Patient-reported outcomes, Health-related quality of life, Reliability, Validity, Rasch analysis, Rating scale

## Abstract

**Background:**

Patient-reported outcome (PRO) data are integral to patient care, policy decision making and healthcare delivery. PRO assessment in pressure ulcers is in its infancy, with few studies including PROs as study outcomes. Further, there are no pressure ulcer PRO instruments available.

**Methods:**

We used gold-standard methods to develop and evaluate a new PRO instrument for people with pressure ulcers (the PU-QOL instrument). Firstly a conceptual framework was developed forming the basis of PU-QOL scales. Next an exhaustive item pool was used to produce a draft instrument that was pretested using mixed methods (cognitive interviews and Rasch Measurement Theory). Finally, we undertook psychometric evaluation in two parts. This first part was item reduction, using PU-QOL data from 227 patients. The second part was reliability and validity evaluation of the item-reduced version using both Traditional and Rasch methods, on PU-QOL data from 229 patients.

**Results:**

The final PU-QOL contains 10 scales for measuring symptoms, physical functioning, psychological well-being and social participation specific to pressure ulcers. It is intended for administration and patients rate the amount of “bother” attributed during the past week on a 3-point response scale. Scale scores are generated by summing items, with lower scores indicating better outcome. The PU-QOL instrument was found to be acceptable, reliable (Cronbach’s alpha values ranging 0.89 - 0.97) and valid (hypothesised correlations between PU-QOL and SF-12 scores (r >0.30) and PU-QOL scales and sociodemographic variables (r <0.30) were consistent with predictions).

**Conclusions:**

The PU-QOL instrument provides a standardised method for assessing PROs, reflecting the domains in a pressure ulcer-specific conceptual framework. It is intended for evaluating patient orientated differences between interventions and in particular the impact from the perspective of patients.

## Background

Chronic wounds are a major health problem and challenge to patients, healthcare professionals and healthcare systems. Pressure ulcers (PUs) are chronic wounds that occur as localised injury to the skin and/or underlying tissue usually over a bony prominence, as a result of pressure, or pressure in combination with shear [1]. They range in size and severity of tissue layer affected, with particularly vulnerable areas being the sacrum, buttocks and heels [2]. With widespread prevalence and incidence in all health settings [3], PUs, often a complication of serious acute or chronic illness, are a health problem associated with increased morbidity [4], mortality [5], healthcare costs and hospitalisation, and identified as a UK National Health Service (NHS) quality indicator [6].

Both PUs themselves and interventions for preventing and treating PUs impact health-related quality of life (HRQL) and can severely compromise all areas of patient functioning [7,8]. Clinical outcomes associated with PU prevention or healing, such as incidence or rate of healing, have been the focus of clinical inquiry; however, due to advances in health outcome measurement, such information alone is no longer sufficient to support progress being made in the PU field [9]. Cochrane reviews highlight the lack of robust evidence for the clinical effectiveness of a majority of PU treatments [10]; resource availability is not based upon health economic evaluation and there is no systematic way of considering patients’ priorities for interventions. Therefore, clinical decision making continues despite being uninformed by high quality studies based on cost-effectiveness and patients’ perspectives.

The field of health is reliant on health outcome measurement to provide a strong evidence-base, incorporating both patient perspectives and cost analyses. In health outcomes research, evaluation of intervention-related outcomes are often undertaken with the help of rating scales, or more recently called patient-reported outcome (PRO) instruments. PRO instruments are increasingly used in clinical studies for measuring outcome variables. In this role, instruments are the central dependent variables which treatment decisions are made. They can be useful tools for evaluating health changes following interventions if they are fit for purpose and accord with international standards for rigorous measurement [9,11].

Patient-based outcome measurement in PUs is in its infancy; few studies have measured PROs and those that have done, have used generic instruments [12]. A PRO instrument specific to PUs could help improve the evidence-base through research assessing effectiveness of PU therapies; facilitate clinician-patient communication and shared decision making; prioritise patient problems and preferences; monitor changes or outcomes of treatment; measure the performance of healthcare providers and services; and be used in clinical audit [13-15].

Our previous work has identified PROs important to people with PUs [7,16,17], established the need for a patient-reported measure of outcomes specific to PUs [12], and developed a provisional version of such a measure (the PU-QOL instrument). The PU-QOL instrument was developed on the basis of a PU-specific HRQL conceptual framework [16] and existing PU and HRQL literature [7]. These sources provided insight into variables important for measurement from the perspective of patients with PUs and were used to generate an exhaustive list of items. The item list (n=122) was transformed into scales intended to define coherent clinically meaningful constructs (scales) consisting of items representing aspects of the continuum of each construct, reflecting the domains within our conceptual framework. This produced a preliminary PU-QOL version which was pre-tested through cognitive interviews with 35 patients with PUs [18], producing a provisional PU-QOL version. Pre-testing identified potential strengths and weaknesses of PU-QOL items, guided decision-making about modifications to items (content and response options) and questionnaire design, and provided early evidence for validity and clinical utility of each PU-QOL scale as reflected by clinically meaningful hierarchical scales, prior to formal psychometric evaluation.

The aim of this study was to provide researchers and clinicians with a comprehensive evaluation of some of the fundamental psychometric measurement properties of the provisional PU-QOL instrument.

## Methods

**Figure 1:**
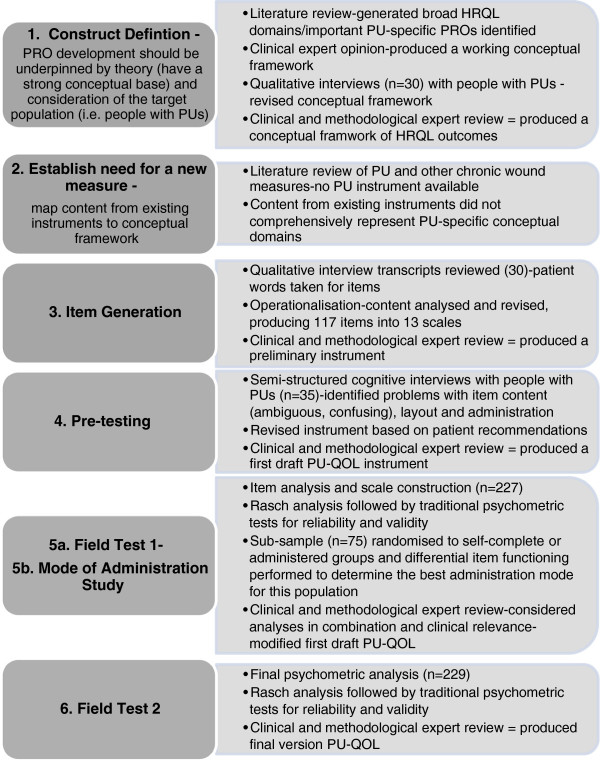
Steps towards developing and evaluating the PU-QOL instrument.

We followed international PRO guidelines [9,19-21] for the development and validation of the PU-QOL instrument (Figure 1). Collaboration was sought from members of the European Pressure Ulcer Advisory Panel (EPUAP) and from 29 acute and primary care NHS organisations around the UK. A UK NHS Research Ethics Committee provided ethical approval and all participants gave written informed consent to participation.

### Field test one design

#### Sample

The first field test was undertaken to construct PU-QOL scales and perform a preliminary psychometric evaluation in a large sample of patients with PUs. Patients from acute and community NHS Trusts around England and Scotland were included if they were aged ≥18 years, with an existing PU of any category, location or duration, and able to provide informed consent to participate. Patients were excluded if they had only moisture lesions, were unconscious, confused, cognitively impaired, deemed ethically inappropriate to approach, did not speak or understand English or unable to provide informed consent.

Eligible patients were purposively sampled, ensuring balanced representation across PU categories (superficial, severe) and skin sites (torso, limb), setting (acute, community), age (<70 years, ≥70) and gender. The ‘rule of thumb’ sample size recommendation for psychometric analyses of new summated scales is five to 10 subjects per item, to reduce the effect of chance [19,22]. Following this recommendation, if the longest potential summated scale was taken (pain containing 11 items), then a 110 patient sample would be required. For the Rasch analysis, a sample of around 250 patients would allow sample selection across the full measurement range; membership to five class interval groups of around 50 patients in each group is suggested [23,24].

#### Rasch analysis

A preliminary psychometric evaluation was performed using both traditional psychometrics in line with proposed US Food and Drug Administration (FDA) criteria [9] and new psychometric methods, Rasch Measurement Theory (RMT) [25]. RMT is increasingly used in the development of PRO instruments [26,27] as it provides a formal method for evaluating scale functioning against a sophisticated mathematical measurement model, the Rasch model [25].

A Rasch analysis, using the Andrich Rating Scale Model [28], was performed using RUMM2030 [29]. The following properties of the provisional PU-QOL version were examined: mode of administration (patient self-completed or researcher administered; *data will be published separately*), scale targeting, item response categories, item series (e.g. item-fit) and response bias, to guide scale construction and identify items with poor psychometric properties for possible elimination. PU-QOL data was tested against model expectations and any deviations were examined to determine whether scales could be improved. Final decisions on item inclusion/exclusion were made according to appraisals of the analyses against measurement criteria (Table 1) and clinical relevance (the extent to which items within proposed scales are clinically cohesive), as opposed to examinations carried out singularly or sequentially.

**Table 1:** Psychometric tests and criteria used in the evaluation of the PU-QOL instrument

**Psychometric property**	**Traditional methods - test and criteria**	**Rasch methods - test and criteria**
**Acceptability and data quality** - Completeness of item- and scale-level data.	● Score distributions (floor/ceiling effects and skew of scale scores)	● Even distribution of endorsement frequencies across response categories (>80%)
	● % of item-level missing data (<10%) [30]	● Low number of persons at extreme (i.e. floor/ceiling) ends of the measurement continuum
	● % of computable scale scores (>50% completed items) [31]	
	● Items in scales rated ‘not relevant’ <35%	
**Scaling assumptions** - Legitimacy of summing a set of items (items should measure a common underlying construct).	● Similar item mean scores [32] and SDs [33]	● Positive residual r between items (<0.30)
	● Items have adequate corrected ITC (ITC ≥0.3) [34]	● High negative residual r (>0.60) suggests redundancy
	● Items have similar ITCs [34]	● Items sharing common variance suggests uni-dimensionality
	● Items do not measure at the same point on the scale	● Evenly spaced items spanning whole measurement range
**Item response categories** - categories in a logical hierarchy.	● NA	● Ordered set of response thresholds for each scale item
**Targeting** - extent to which the range of the variable measured by the scale matches the range of that variable in the study sample.	● Scale scores spanning entire scale range	● Person-item threshold distribution: person locations should be covered by items and item locations covered by persons when both calibrated on the same metric scale [35]
	● Floor and ceiling (proportion sample at minimum and maximum scale score) effects should be low (<15%) [36]	
	● Skewness statistics should range from −1 to +1 [37]	● Good targeting demonstrated by the mean location of items and persons around zero
	● No published criteria for item level targeting	
**Reliability**		
Internal consistency - extent to which items comprising a scale measure the same construct (e.g. homogeneity of the scale).	● Cronbach's alphas for summary scores (adequate scale internal consistency is ≥0.70 [22]	● High person separation index >0.7 [38]; quantifies how reliably person measurements are separated by items
	● Item-total r between +0.4 and +0.6 indicate items are moderately correlated with scale scores; higher values indicate well correlated items with scale scores [22]	
		● Power-of-tests indicate the power in detecting the extent to which the data do not fit the model [24]
		● Items with ordered thresholds
*Test-retest reliability - stability of a measuring instrument.	● Intra-class r coefficient >0.70 between test and retest scores [11]	● Statistical stability across time points (no uniform or non-uniform item DIF (p=>0.05 or Bonferroni adjusted value))
	● Pearson r: >0.7 indicates reliable scale stability	
**Validity**	● Involves accumulating evidence from different forms	
Content validity - extent to which the content (items) of a scale is representative of the conceptual construct it is intended to measure.	● Consideration of item sufficiency and the target population	● Clearly defined construct
	● Qualitative evidence from individuals for whom the measure is targeted, expert opinion and literature review (e.g. theoretical and/or conceptual definitions) [9].	● Validity comes from careful item construction and consideration of what each item is meant to measure, then testing against model expectations
Construct validity		
i) Within-scale analyses - extent to which a distinct construct is being measured and that items can be combined to form a scale score.	● Cronbach alpha for scale scores >0.70	● Fit residuals (item-person interaction) within given range +/−2.5
	● ITC >0.30	
	● Homogeneity coefficient (IIC mean and range >0.3)	● Non-significant chi square (item-trait interaction) values
	● Scaling success	● No under- or over-discriminating ICC
		● Mean fit residual close to 0.0; SD approaching 1.0 [39]
		● Person fit residuals within given range +/−2.5
Measurement continuum - extent to which scale items mark out the construct as a continuum on which people can be measured.	● NA	● Individual scale items located across a continuum in the same way locations of people are spread across the continuum [26]
		● Items spread evenly over a reasonable measurement range [40,41]. Items with similar locations may indicate item redundancy
Response dependency –response to one item determines response to another.	● NA	● Response dependency is indicated by residual r >0.3 for pairs of items [40,41]
ii) Between scale analysis		
Criterion Validity - hypotheses based on criterion or ‘gold standard’ measure.	● There are no true gold standard HRQL [42], PU-specific or chronic wound-specific measures available [12]	● NA
*Convergent validity - scale correlated with other measures of the same/ similar constructs.	● Moderate to high r predicted for similar scales; criteria used as guides to the magnitude of r, as opposed to pass/fail benchmarks (high r >0.7; moderate r=0.3-0.7; low r <0.3) [43]	● NA
*Discriminant validity – scale not correlated with measures of different constructs	● Low r (<0.3) predicted between scale scores and measures of different constructs (e.g. age, gender)	● NA
*Known groups differences - ability of a scale to differentiate known groups	● ^Generate hypotheses (based on subgroups known to differ on construct measured) and compare mean scores (e.g. predict a stepwise change in PU-QOL scale scores across 3 PU severity groups and that mean scores would be significantly different)	● Hypothesis testing (e.g. clinical questions are formulated and the empirical testing comes from whether or not data fit the Rasch model)
	● Statistically significant differences in mean scores (ANOVA)	
*Differential item functioning (item bias) - The extent of any conditional relationships between item response and group membership.	● NA	● Persons with similar ability should respond in similar ways to individual items regardless of group membership (e.g. age) [44]
		● Uniform DIF - uniformity amongst differences between groups
		● Non-Uniform DIF - non-uniformity amongst differences between groups; can be considered at 1% (Bonferroni adjusted) and 5% CIs

Table adapted from [35,45]; *Additional tests performed for field test two; ^The PU *HRQL* literature is not well established, therefore was limited for identifying clinical parameters to formulate known groups; *NA* No test for particular psychometric property; *SD* Standard deviation; *ITC* Item total correlation; *IIT* Inter-item correlation; *ICC* Item characteristic curve; *r* correlation; *ANOVA,* Analysis of variance; *DIF* Differential item functioning; *CI* Confidence interval.

#### Traditional analysis

The 10 Rasch constructed scales underwent a preliminary psychometric evaluation using traditional psychometric tests [9,11,22] for: acceptability, scaling assumptions, reliability, and validity. SPSS 15.0 software was used for these analyses. Psychometric tests and criteria are summarised in Table 1.

### Field test two design

#### Sample

The second field test was undertaken to perform a comprehensive psychometric evaluation of the final (10 scale/83-item) PU-QOL in a large independent sample of patients with PUs (eligibility criteria and methods as for field test 1.1). A sample of around 250 patients would provide sufficient participants to estimate test-retest reliability; correlations at levels expected in test-retest situations (e.g. r >= 0.80) can be estimated with reasonable precision (95% confidence intervals of ±0.1) with relatively few subjects [46,47].

#### Rasch analysis

A Rasch analysis was performed on all 10 PU-QOL scales. In addition to the properties examined in for field test 1, differential item functioning (DIF) was also assessed. DIF occurs when people from different groups (e.g. gender) with the same latent trait (e.g. pain) have a different probability of giving a certain response to an item [44]. Groups to be studied were selected based on theoretical considerations about whether or not the construct measured by each PU-QOL scale was hypothesised to have the same conceptual meaning across groups.

#### Traditional analysis

The final PU-QOL version underwent traditional psychometric analyses as described in for field test 1. Additional tests for reliability (test re-test) and validity, including both within- and between-scales testing (convergent, discriminant, known groups) were undertaken (Table 1). To minimise respondent burden, the SF-12v2 Acute, English (UK) version was used [48] to examine convergent validity.

## Results

### Field-test one: scale construction and preliminary psychometric evaluation

#### Sample

The first field test screened 989 patients from 21 hospitals, 10 community services and one hospice. Of those screened, eligibility was assessed for 787 (79.6%); 416 were considered eligible (52.9%); and of those eligible, 287 (69.0%) consented to participate; however, 60 were excluded from analysis as they self-completed the PU-QOL (data on the self-completed sample will be published elsewhere). Cognitive impairment was the main reason for ineligibility (38.8%). Table 2 presents the sample characteristics.

**Table 2:** Participant characteristics

	** Field test 1**	** Field test 2**
**Characteristics**	** Range (Mean, SD)**	** Range (Mean, SD)**
**Age**	24 - 98 (72, 13.5)	20 - 103 (71.3, 16.5)
**Gender**	** Total n (%)**	** Total n (%)**
Total	n=227	n=229
Male	90 (39.6)	119 (52.0)
Female	137 (60.4)	110 (48.0)
**Ethnicity**		
White	223 (98.2)	227 (99.1)
Asian	1 (0.4)	2 (0.9)
Black/African	2 (0.4)	0
Chinese	0	0
Not stated	1 (0.4)	0
**Setting**		
Hospital (surgery)	99 (43.6)	62 (27.1)
Hospital (medicine)	21 (9.3)	74 (32.3)
Community	107 (47.1)	88 (38.4)
**PU severity**		
Category 1	38 (10.6%)	76 (18.1%)
Category 2	144 (40.2%)	170 (40.5%)
Category 3/4	153 (42.7%)	170 (40.5%)
Missing	1 (0.3%)	4 (0.9%)
**PU risk classification**		
Short-term risk	39 (17.2)	36 (15.7)
Medium to long-term risk	71 (31.3)	87 (38.0)
On-going long-term risk	116 (51.1)	103 (45.0)
Missing	1 (0.4)	3 (1.3)
**Marital status**		
Single	59 (26.0)	71 (31.0)
Married	85 (37.5)	77 (33.6)
Cohabiting	81 (35.7)	75 (32.8)
Missing	2 (0.8)	6 (2.6)
**Living arrangements**		
Live alone	84 (37.0)	86 (37.6)
Cohabit with carer	63 (27.8)	51 (22.3)
Cohabit with other	61 (26.9)	48 (20.9)
Missing	19 (8.4)	44 (19.2)
**Education**		
No formal education	129 (56.8)	125 (54.6)
GCSE or equivalent	39 (17.2)	40 (17.5)
A-Level or equivalent	25 (11.0)	16 (6.9)
Degree or higher	15 (6.6)	21 (9.2)
Missing	19 (8.4)	27 (11.8)

#### Rasch analysis: item reduction and scale formation

The first psychometric evaluation produced a 10-scale instrument (Table 3). The Rasch analysis detected important limitations of the PU-QOL scales, resulting in modifications. It detected that the four-category item scoring function did not work as intended for multiple items. For those items where the response categories were working as intended, thresholds were close to being disordered; people had difficulty distinguishing between ‘a little bother’ and ‘quite a bit of bother’ categories. This provided good evidence that items would benefit from fewer response categories. All scale items were subjected to a post hoc rescoring by collapsing adjacent categories. Re-analysis demonstrated that all thresholds were now correctly ordered, producing scales with three categories (0 = no bother, 1 = little bother, 2 = a lot of bother).

**Table 3:** Summary of preliminary PU-QOL instrument psychometric analysis, field test 1

**Scale (No. of items)**	**Rasch analysis**					**Traditional psychometric analysis**		
	**Items with disordered thresholds**	**Item locations logits range**	**Fit statistics fit residuals outside +/−2.5**	**Items with Chi square probability significance ≥0.001**	**Person separation index**	**Cronbach alpha**	**Range IIC**	**Scaling assumptions corrected ITC**
Pain (8)	5	-0.94 − 0.80	0	4	0.78	0.89	0.24 – 0.66	0.53 – 0.70^
Exudate (8)	4	-0.51 − 0.48	0	0	0.59	0.92	0.40 – 0.86	0.56 – 0.84^
Odour (6)	2	-1.47 − 0.60	0	0	0.62	0.96	0.74 – 0.91	0.83 – 0.92^
Sleep (6)	3	-0.54 − 0.31	0	0	0.62	0.92	0.48 – 0.84	0.67 – 0.86^
Vitality (3)	0	-0.48 − 0.44	0	0	0.03	n/a	n/a	n/a
Movement/mobility (11)	4	-0.33 − 0.48	0	0	0.58	0.93	0.23 – 0.91	0.67 – 0.80^
ADL (9)	8	-0.54 − 0.57	0	0	0.29	0.95	0.41 – 0.90	0.58 – 0.90^
Emotional well-being (17)	4	-1.15 − 1.46	1	0	0.82	0.93	0.24 – 0.79	0.54 – 0.76^
Appearance & self- consciousness (7)	4	-0.83 − 0.65	0	0	0.56	0.90	0.41 – 0.75	0.60 – 0.79^
Participation (9)	4	-0.56 − 0.54	0	0	0.65	0.96	0.53 – 0.89	0.73 – 0.90^

*IIC* inter-item correlation; ^Range item-total correlation (ITC).

Targeting between the distribution of person measurements and item locations indicated that the samples were adequate for examining the scales but the scales were suboptimal for measuring the sample. Significant ceiling effects indicated that scales might provide limited information about people at the extremes of the sample distribution (those with least disability/impairment). However, the location ordering of scale items was clinically sensible, providing evidence towards construct validity. Some items had notable criterion failures: fit residuals outside +/−2.5; high chi-squared values with significant p-value, and significantly under- or over-discriminating item characteristic curves (Table 3). Few items exceeded +/−0.3 residual correlations, indicating that item responses are independent of each other and no redundant items. Departures from item fit expectation were considered in combination and guided item removal. Person separation index values indicated good to reasonable reliability for scales distinguishing between responders on each scale variable (Table 3).

#### Traditional analysis

A preliminary psychometric evaluation against traditional psychometric criteria supported the PU-QOL scales as reliable and valid measures of PU-symptoms, physical and social functioning, and psychological well-being. Briefly, data quality was high (scale scores were computable for 93–99.6% of respondents) and scaling assumptions were satisfied (similar mean item scores, corrected item-total correlations ranged 0.53-0.92). Scale-to-sample targeting was good (scale scores spanned the scale range but were notably skewed for three scales (values outside +/−1.0), mean scores were near scale mid-points for 67% of scales, and ceiling effects were negligible; however floor effects exceeded the 15% criterion for two scales. Reliability was high as demonstrated by Cronbach’s alpha values (range 0.89-0.96; Table 3). The item-total correlations, alpha coefficient and homogeneity coefficient (inter-item correlation mean and range; Table 3) provide evidence towards the internal construct validity of PU-QOL scales.

### Field-test two: final psychometric evaluation

#### Sample

The second field test involved a comprehensive psychometric evaluation of the final (10 scale/83-item) PU-QOL, using RMT and traditional psychometric methods. A total of 879 patients were screened of whom eligibility was assessed for 717 (81.6%); 391 were considered eligible (54.5%); and of those eligible, 231 (59.1%) consented to participate; however two were excluded from analysis (one patient died; one patient was recruited twice). Table 2 presents the sample characteristics.

#### Rasch analysis

The measurement properties of PU-QOL scales were largely supported as demonstrated through items that mapped out continua of increasing intensity and located items along those continua in a clinically sensible order. Scale items work together to define single variables, albeit, some item misfit and local dependence (Table 4). DIF was demonstrated in three items (e.g. items ‘difficulty standing for long periods’ and ‘limited in ability to go up and down stairs’ from the mobility scale; Table 4), however deviations from model expectations were marginal, suggesting item performance across the four clinical subgroups is stable and that these groups can be measured on a common ruler.

**Table 4:** Summary of PU-QOL Rasch analysis, field test 2

**Scale (No. of items)**	**Disordered thresholds**	**Item locations logits range**	**Fit statistics fit residuals outside +/−2.5**	**Items with Chi square probability significance ≥0.001**	**Person separation index**	**DIF age**		**DIF gender**		**DIF HC setting**	
						**Uni**	**Non**	**Uni**	**Non**	**Uni**	**Non**
Pain (8)	0	-1.11 − 1.03	0	0	0.72	0	0	0	0	0	0
Exudate (8)	1	-0.75 – 0.84	1	1	0.69	0	0	0	0	0	0
Odour (6)	0	-1.31 – 0.91	0	0	0.66	0	0	0	0	0	0
Sleep (6)	0	-0.91 – 0.45	1	1	0.62	0	0	0	0	0	0
Mobility and movement (9)	2	-0.46 – 0.57	0	0	0.42	0	0	0	0	2	0
Activity (8)	4	-0.30 – 0.56	0	0	0.27	0	0	0	0	0	0
Vitality (6)	0	-0.50 – 0.81	0	0	0.38	1	0	0	0	0	0
Emotional well-being (15)	2	-1.48 – 2.44	0	0	0.86	0	0	0	0	0	0
Self-consciousness (7)	0	-1.27 – 1.02	0	0	0.58	0	0	0	0	0	0
Participation (9)	7	-0.91 – 1.00	0	0	0.57	0	0	0	0	0	0

DIF differential item functioning; HC healthcare; Uni uniform DIF; Non non-uniform DIF.

The Rasch analysis detected some important limitations; the three-category scoring function did not work as intended for some scale items, indicated by disordered thresholds (e.g. items ‘walking slowed’ and ‘limited in ability to walk’ from the mobility scale; items ‘regular activities’ and ‘jobs around the house’ from the activity scale), and targeting problems emerged. Inspection of threshold distributions demonstrated sub-optimal targeting of PU-QOL scales to the study sample for most scales (items did not span the full range of the patient sample, indicating that measurement could be improved at the extreme ends of some scales; Table 4. The largest frequency of respondents was often at the ceiling of scale ranges (least bother). Ideally, there should be a good match between the scale and sample ranges, with people falling within the range of the items. As sample sizes were small for some scales (e.g. removing people with no odour bother resulted in a sample of 27 for analysis), it was deemed premature to make major modifications to items and the scoring function without additional empirical evidence.

#### Traditional analysis

The traditional psychometric evaluation supported the PU-QOL scales as reliable and valid measures of PU-symptoms, physical and social functioning, and psychological well-being. Total scores could be computed for most people (computable scale scores ranged 95.6-99.6%), implying good data quality. Scaling assumptions were satisfied (corrected item-total correlations ranged 0.51-0.94). All item-own-scale correlations were high (corrected item-total correlations ranged 0.525-0.920; Table 5) and satisfied recommended criteria (> 0.3), thus providing support that items within scales measured a common underlying construct. Corrected item-total correlation >0.3 indicated that items within scales contained a similar proportion of information. Scale-to-sample targeting was reasonable: scale scores spanned the scale ranges but were notably skewed for exudate odour and self-consciousness scales (value outside +/−1.0); mean scores were near scale mid-points for only pain, sleep and mobility scales, however due to many people responding at the floor (lowest score), this finding is expected; and ceiling effects were negligible, however floor effects exceeded the 15% criterion for exudate, odour, vitality, and appearance and self-consciousness scales.

**Table 5:** Summary of PU-QOL traditional psychometric analysis, field test 2

**Scale (No. of items)**	**Internal consistency Cronbach’s alpha**	**IIC**	**Scaling assumptions-corrected ITC**	**Test retest reproducibility**			**Convergent validity**		**Discriminant validity**	
				**ICC consistency**	**ICC absolute**	**Correlation**	**Related SF12 scale r**^**1**^	**PU-QOL HRQL item r**^**1 **^**(n)**	**Gender R**^**2 **^**(n)**	**Age r**^**2 **^**(n)**
Pain (8)	0.89	0.24 – 0.66^	0.53 – 0.70^	0.80	0.81	0.80	0.48^b^	0.38^b^ (206)	0.13^b^ (214)	0.11^b^ (214)
Exudate (8)	0.91	0.32 – 0.72^	0.51 – 0.75^	0.62	0.63	0.62	n/a	0.25^a^ (216)	0.08^b^ (225)	-0.14^b^ (224)
Odour (6)	0.97	0.72 – 0.93^	0.79 – 0.94^	0.68	0.68	0.70	n/a	0.20^a^ (217)	0.05^b^ (228)	-0.14^b^ (227)
Sleep (6)	0.92	0.49 – 0.81^	0.68 – 0.85^	0.82	0.82	0.82	n/a	0.32^b^ (171)	0.21^b^ (178)	0.10^b^ (178)
Vitality (6)	0.90	0.49 – 0.90^	0.63 – 0.90^	0.74	0.74	0.74	0.36^b^	0.52^b^ (135)	0.03^b^ (137)	-0.16^b^ (137)
Movement/Mobility (9)	0.93	0.23 – 0.91^	0.67 – 0.80^	0.87	0.86	0.88	-0.50^b^	0.39^b^ (37)	0.04^b^ (39)	0.22^b^ (39)
ADL (8)	0.95	0.41 – 0.90^	0.58 – 0.90^	0.87	0.87	0.87	-0.38^b^	0.35^b^ (48)	-0.05^b^ (49)	-0.19^b^ (49)
Emotional well-being (15)	0.94	0.24 – 0.79^	0.54 – 0.76^	0.83	0.82	0.83	-0.44^b^	0.58^b^ (133)	0.16^b^ (135)	-0.15^b^ (135)
Appearance & self-consciousness (7)	0.89	0.37 – 0.79^	0.62 – 0.76^	0.81	0.81	0.81	-0.40^b^	0.50^b^ (176)	0.23^b^ (179)	-0.03^b^ (178)
Participation (9)	0.93	0.36 – 0.88^	0.60 – 0.86^	0.63	0.64	0.63	-0.52^b^	0.51^b^ (75)	0.01^b^ (76)	-0.29^b^ (76)

*IIC* inter-item correlation; ^Range item-total correlation (ITC); ^1^Spearman correlation; ^2^Pearson correlation; ^a^Correlations falling outside of the predicted range; ^b^Correlations consistent with predictions.

Reliability was high as demonstrated by Cronbach’s alpha values for all PU-QOL scales exceeding the standard criterion of 0.7 (Table 5). Item–total correlations ranged 0.525-0.920, fulfilling the recommended criteria (>0.3). Test-retest correlations for 8/10 scales exceeded 0.7; two scales had correlations below the recommended criteria, but marginally (Table 5), thus mostly fulfilling the recommended minimum criteria and indicating good scale stability.

Evidence of internal construct validity was supported by moderate to high item-total correlations; high Cronbach’s coefficient alphas; and moderate to high inter-item correlations (means >0.48; ranges 0.226-0.934; Table 5), indicating that each PU-QOL scale measures a single construct. Hypothesised correlations between PU-QOL and related SF-12 scales were consistent with predictions (Table 5), thus providing support that scales measure what they intend to measure; moderate to high correlations (r >0.30) were predicted. Correlations between PU-QOL scales and sociodemographic variables (age, gender) were consistent with predictions (r <0.30; Table 5), suggesting responses to scales are not biased by age or gender. Hypothesised group differences were as predicted for scales: exudate, odour, vitality, daily activities, emotional well-being, and self-consciousness, with significant step increases in mean scores observed by PU severity groups. In contrast, there was no step increase in mean scores for scales: pain, sleep, mobility and movement, and participation. Apart from the sleep scale, the mean score on outcomes for category 1 PU severity was lower than category 3/4 severity, suggesting that HRQL outcomes are worse for people with severe PUs compared to those with superficial category 1 PUs. It is important to note that category 1 PUs had small samples (range 4–14 patients) therefore known groups results are considered preliminary.

#### Final PU-QOL Instrument

The final PU-QOL is a self-report instrument, comprising of 10 scales. These include three symptom (pain (8 items), exudate (8 items), odour (6 items)), plus an itchiness item; four physical functioning (sleep (6 items), movement and mobility (9 items), daily activities (8 items), vitality (5 items)); two psychological well-being (emotional well-being (15 items), self-consciousness and appearance (7 items)); and one social participation scale (9 items). It is intended for administration where patients rate the amount of “bother” attributed (e.g. “During the past week, how much have you been bothered by…?”) on a 3-point response scale (e.g. 0=not at all - 2=a lot). Scale scores are generated by summing items and then transforming to a 0–100 scale. High scores indicate greater patient bother.

## Discussion

The PU field requires a strong evidence-base that incorporates health outcome measurement from the patient perspective. To fully capture and quantify the patients’ viewpoint, appropriately constructed and validated instruments are required. The PU-QOL instrument consists of 10 scales for measuring symptoms and physical, psychological, and social functioning specific to PUs. This is the first outcome measure reflecting PU-specific conceptual HRQL domains; content that differs from other chronic wound-specific instruments [12], and provides a framework for designing future research that consequently improves the quality of research in the field by inclusion of PU-specific PROs.

Scale development and item reduction were primarily guided by RMT. RMT provides a powerful framework to guide scale construction by detecting items deviating from model expectations with the intention of improving scale attributes. Evidence from RMT was used to understand why some scale items were not working and to pin point where improvements could be made. However, final decisions on item inclusion were made according to appraisals of the analyses of the observed data against measurement criteria and clinical relevance, as opposed to examinations carried out singularly or sequentially.

The final psychometric evaluation demonstrated that PU-QOL scales mostly satisfy criteria for acceptability, reliability and validity, in line with recommended FDA guidelines for measurement [9]. However, the Rasch analysis detected targeting problems despite attempts to sample a wide variety of patients with PUs drawn across settings. Targeting is justified for the exudate and odour scales as not all patients have these problems; it is clinically reasonable that these people fall outside the scale range. Importantly, where people have symptom bother, there needs to be items within the scales that discriminate symptom bother, and in this instance, the symptom scales perform this function. For the remaining scales, targeting could be improved by developing items that span a wider measurement range, and in the process, maximise the potential of the PU-QOL to detect change. Extending the measurement range can be achieved without affecting the scales as they stand, because the item locations are calibrated relative to each other. Important to note, scale scores for >65% of the samples were within the best performing part of all scales. For example, the pain scale items spread 2-logits compared to a person spread of 7 logits, indicating suboptimal targeting. But for the majority of people in the sample, the measurement range distribution was within the range where most people lay, indicating good pain scale performance.

Given the heterogeneity of the population with PUs, further work is required to ensure that the PU-QOL scales fit the needs of all people with PUs including patients with superficial PUs. Appropriateness of PU-QOL’s use in individual decision-making needs investigation; strengthening the measurement precision could improve the PU-QOLs ability to detect differences in HRQL outcomes between people with different PU severity. This is important for making inferences from future research using the PU-QOL. However, one consideration is that during field testing, as is standard practice, patients received some form of treatment for their PU; information that was not collected (e.g. amount of analgesia). Therefore, the true impact of PUs may not have been captured (lower severity represented in the sample due to treatment effect) and be the reason for, at least in part, mistargeting and misrepresentation of known groups testing. In actual fact, PUs appear to cause patients more bother (as indicated from the qualitative work) than was represented but good care received lowered PU impact in the sample. This is a methodological issue in this area. Finally, the three-category scoring function did not work as intended for some scale items and requires exploration. The above limitations do not preclude use of the PU-QOL instrument. PU-QOL scales can be included as one outcome measure, amongst others, for group comparisons in future PU research (e.g. clinical trials) on the proviso that studies have built in a parallel psychometric analysis to indicate the performance (psychometric evaluation) of the scales in future samples.

The final Rasch analyses provides an initial evidence-base for future testing to improve the PU-QOL scales and to establish the extent that psychometrically sound scales have been developed. Future scale developments can be empirically driven; the distribution of item locations highlight where ‘gaps’ in the measurement continuum are (fill notable distances in item locations with items, particularly those representing superficial PU impact and extend the measurement range at the extreme ends of the continuum). The process of modifying a newly developed instrument is part of an evolving, on-going measurement process intended to strengthen the hypothesised conceptual relationships with empiric evidence [49]. The usefulness of new measures is therefore demonstrated by multiple applications in different studies (accumulative body of evidence to support scale measurement properties). Future research will investigate the sensitivity of PU-QOL scales to change and responsiveness, and develop an instrument to enable economic evaluation. Development of proxy measures and language translations are needed given the high prevalence of cognitively impaired patients with PUs.

The PU-QOL instrument is intended for administration, following a user manual, with adults across the range of PU severity and type (location and duration) and UK acute and community healthcare settings. Scales can be selected depending on the nature of the research and scale items are summed to produce scores. The PU-QOL can be used for: effectiveness intervention research where improvement and/or deterioration in HRQL is measured; promoting patient-clinician communication (i.e. flag issues); informing changes to treatment; facilitating priority setting and patient care and PU management decisions and assessing the care given from the patient’s perspective. Currently, the PUQOL is most appropriate for people with severe PUs, as demonstrated by a lack of items to represent people with little or no bother due to PUs. The exudate and odour scales are not intended for people with superficial category 1 PUs. Electronically defined ‘skip’ questions would assist in selecting scales and items relevant to each individual’s circumstance.

As the PU-QOL was developed and evaluated in the UK, the validity and reliability are characteristics of the instrument for a specific population (i.e. UK nationals) and should therefore be re-evaluated for a new population. A language translation or cross-cultural adaption may be required to ensure that the PU-QOL is appropriate for cultures, languages and ethnic groups outside the UK (see the PU-QOL instrument website for guidance on language translation and cross-cultural adaptation processes: http://ctru.leeds.ac.uk/Skin).

This research highlights the importance of fully testing instruments before clinicians and researchers apply them. It highlights the value of item-level analyses, not typically undertaken, that identified problems with the PU-QOL scales not detected by standard tests of scale reliability and validity. It also demonstrated that small iterative steps, using mixed methods in an interactive way, rather than the traditional three stage approach to PRO development (i.e. qualitative work to generate constructs and content, pre-testing and psychometric evaluation) may be beneficial, particularly at early content and scale format/design to understand and resolve instrument issues early in the development process. Both qualitative and empirical findings should be used to inform subsequent work and to make improvements to scales. Uniformity of research approaches for PRO development could lead to consistency in health measurement and the inclusion of mixed methods as well as the more sophisticated psychometric methods, such as RMT in accepted international guidelines.

### Conclusions

This study makes important contributions to the PU and wider health measurement fields. The findings demonstrate that mixed methods, including RMT were beneficial for developing a new PRO instrument specific for PUs; a methodology that can be applied for further development of the PU-QOL as well as PROs in other health areas. The PU-QOL instrument provides a means for the comprehensive assessment of PU impact and for quantifying the benefits of PU interventions from the patients perspective; thus far lacking in the area. A scientifically rigorous PRO measurement needs to become more commonplace in the PU field so that the goal of PU management can be to enhance and maintain the HRQL of people with PUs. Subject to further development, PU-QOL is a tool with which to evaluate whether PU treatments and the healthcare given achieve this; outcomes that are ultimately best judged by patients themselves.

## Competing interests

The authors declare they have no completing interests.

## Authors’ contributions

CG contributed to study concept and design, acquisition of study data, analysis and interpretation of results, and preparation of the final manuscript. JB contributed to study design, interpretation of results and preparation of the final manuscript. SC contributed to study design, psychometric analysis, interpretation of data, and preparation of the final manuscript. DL contributed to study design, interpretation of data and provided methodological expertise. MB, SCo, CD, EM, EAN, NS and LW contributed to interpretation of results and preparation of the final manuscript, and provided clinical expertise throughout the duration of the study. JN contributed to study concept and design, interpretation of results and preparation of the final manuscript. All authors read and approved the final manuscript.
